# Gastrointestinale Stromatumoren: Wo stehen wir?

**DOI:** 10.1007/s00292-024-01318-5

**Published:** 2024-04-08

**Authors:** Eva Wardelmann, Anna Kuntze, Artem Voloshin, Sandra Elges, Marcel Trautmann, Wolfgang Hartmann

**Affiliations:** Gerhard-Domagk-Institut für Pathologie, Universitätsklinikum Münster, Albert-Schweitzer-Campus 1, 48149 Münster, Deutschland

**Keywords:** Risikoklassifikation, Molekulare Typisierung, Succinatdehydrogenase, Genetische Prädisposition, Syndromale Krebserkrankung, Risk assessment, Molecular typing, Succinate dehydrogenase, Genetic predisposition, Syndromic cancer

## Abstract

Gastrointestinale Stromatumoren (GIST) stellen seit über 20 Jahren ein Paradigma für die zielgerichtete Therapie mit Tyrosinkinaseinhibitoren dar. Eine elementare Voraussetzung für eine mögliche neoadjuvante oder adjuvante Behandlung bei lokalisierten GIST bzw. eine additive Therapie bei metastasierten GIST ist die molekulare Typisierung der Tumoren, idealerweise bereits bei Erstdiagnose. Zudem ist auf die Möglichkeit einer hereditären oder syndromalen Prädisposition zu achten, da sich hieraus auch therapeutische Konsequenzen und eine andere Nachsorgestrategie ergeben.

## Abstract (en)

For more than 20 years gastrointestinal stromal tumors (GIST) have been a paradigm for a targeted treatment with tyrosine kinase inhibitors. A fundamental prerequisite for a neoadjuvant or adjuvant treatment of localized GIST or an additive treatment of metastatic GIST is the molecular typing of tumors, ideally at the initial diagnosis. In addition, the possibility of a hereditary or syndromic predisposition must be considered because this results in consequences for the treatment and a different follow-up strategy.

## Lernziele

Nach der Lektüre dieses Beitrags …

- kennen Sie die unterschiedlichen Subtypen der gastrointestinalen Stromatumoren (GIST).
- wissen Sie, welche immunhistochemischen Marker Ihnen die Diagnose ermöglichen.
- können Sie schon anhand des Subtyps erste prognostische Vorhersagen des jeweiligen GIST treffen.
- können Sie die klinisch tätigen Kollegen bezüglich der weiteren zu favorisierenden Therapie beraten.
- sind Sie in der Lage, syndromale und hereditären Subtypen zu erkennen.

## Einleitung

Gastrointestinale Stromatumoren (GIST) stellen die häufigsten **mesenchymalen Tumoren**

mesenchymalen Tumoren

im Magen-Darm-Trakt dar. Ihre jährliche Inzidenz beträgt in Deutschland ca. 6 bis 10 Neuerkrankungen/100.000 Einwohner, was etwa 8000 Neuerkrankungen/Jahr entspricht [1]. Die **molekulare Diagnostik**

molekulare Diagnostik

dieser Tumoren wurde schon frühzeitig als hoch relevant für Diagnostik, Prognose und Prädiktion erkannt und etabliert. Die schon damals gewonnenen Erkenntnisse zu **sekundären Resistenzen**

sekundären Resistenzen

nach Verabreichung von Tyrosinkinaseinhibitoren (TKI) deckten Mechanismen auf, die heute paradigmatisch auch bei anderen mit TKI behandelten Tumorentitäten beobachtet werden [2]. Der zunehmende Einsatz der Tiefensequenzierung hat zudem alternative Pathomechanismen bei GIST, die jenseits von *KIT*- oder *PDGFRA*-Mutationen liegen, aufgedeckt.

## Epidemiologie

Die meisten GIST treten im höheren Lebensalter auf, mit einem Altersgipfel zwischen der 6. und 7. Dekade. Gleichwohl können sich GIST in allen Altersstufen und insbesondere bei Kindern entwickeln. Etwa zwei Drittel der Tumoren finden sich im **Magen**

Magen,

bis zu 30 % im **Dünndarm**

**Figure 1:**
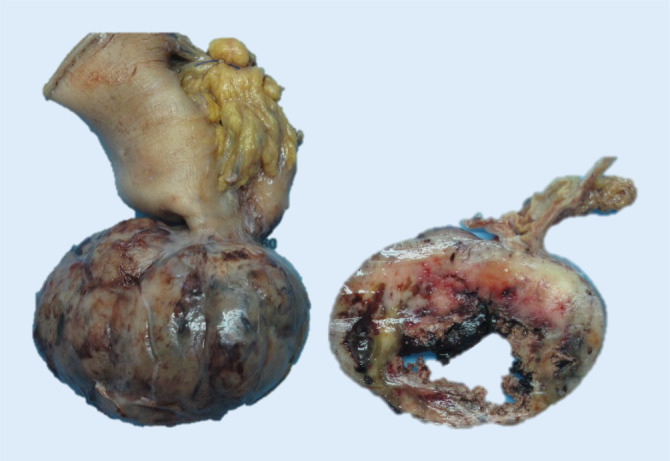

Dünndarm

(makroskopisches Beispiel in Abb. 1). Mit etwa 5 % wird ihre Häufigkeit im **Rektum**

Rektum

angegeben, während das restliche Kolon nur exzeptionell selten betroffen ist [1]. Seltener werden GIST ohne eindeutigen Bezug zum tubulären Gastrointestinaltrakt beobachtet und von einigen Gruppen als „extragastrointestinale (E-)GIST“ bezeichnet, ohne dass diese Möglichkeit bislang sicher histogenetisch bewiesen worden wäre. In etwa 1 % der Fälle kommen GIST primär im **Ösophagus**

Ösophagus

vor [3].

### Merke

Gastrointestinale Stromatumoren kommen am häufigsten im Magen vor.

## Immunhistochemisches Markerprofil

**Figure 2:**
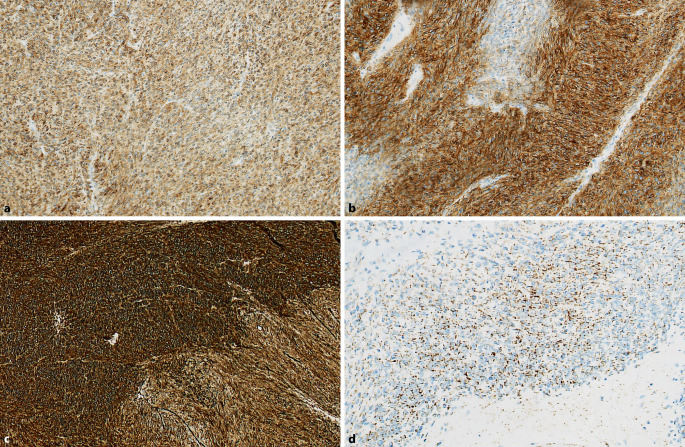

Der erste Antikörper, der die Diagnostik von GIST als eigene Entität ermöglicht hat, ist der KIT-Rezeptor (CD117; Abb. 2a). Bis zu 90 % aller GIST exprimieren diesen Marker häufig auch kräftig, wobei membranäre, zytoplasmatische und dotförmige Färbemuster einzeln oder kombiniert auftreten. Ein noch sensitiverer Antikörper insbesondere bei GIST ohne *KIT*-Mutation ist DOG1 („detected on GIST1“), ein Kalziumkanalprotein (Abb. 2b). Die absolute Ausnahme sind DOG1-negative GIST (≪ 1 %). Diagnostisch hilfreich kann außerdem der Nachweis von CD34 sein; dieser gelingt in 70–80 % der GIST ([4]; Abb. 2c). Die **Proliferationsaktivität**

Proliferationsaktivität

kann mithilfe von Ki67 dargestellt werden, auch wenn der Ki67-Index bislang in keinen Risikoscore für diese Tumoren aufgenommen wurde. Ein weiterer relevanter immunhistochemischer Marker ist die **Succinatdehydrogenase-Isoform B**

Succinatdehydrogenase-Isoform B

(SDHB), die sich als zytoplasmatisches granuläres Protein nachweisen lässt (Abb. 2d). Bei einer inaktivierenden SDH-Mutation (weiteres zum SDH-defizienten GIST s. Abschn. „Sporadische und hereditäre SDH-defiziente GIST“) fällt diese Expression in den Tumorzellen unabhängig davon, welche der 4 SDH-Isoformen inaktiviert ist, aus. Der Antikörper eignet sich daher hervorragend für Screeningzwecke [5].

### Merke

Der sensitivste Marker für gastrointestinale Stromatumoren ist DOG1 gefolgt von CD117.

## Risikoklassifikation

Die gängigste Risikoklassifikation stellt nach wie vor die von Miettinen und Lasota 2006 publizierte Einteilung dar. Hier werden Lokalisation, Größe und Mitosenzahl/5 mm^2^ als Prognoseparameter berücksichtigt ([1]; Tab. 1). Es sind 5 Risikogruppen vorgesehen: kein nennenswertes, sehr niedriges, niedriges, intermediäres und hohes Risiko. Außerdem sollte berücksichtigt werden, dass eine prä- oder intraoperative **Tumorruptur**

Tumorruptur

das Rückfallrisiko drastisch erhöht [6]. Dieser zusätzliche Parameter ist in die ebenfalls hilfreichen **„contour maps“**

„contour maps“

von Joensuu et al. [7] integriert.

**Table 1:** 

Gruppe	Größe	Mitosenzahl/5 mm^2^ ^*^	Lokalisation			
			Magen	Jejunum/Ileum	Duodenum	Rektum
1	≤ 2 cm	≤ 5	ø	ø	ø	ø
2	> 2–5 cm	≤ 5	Sehr niedrig	Niedrig	Niedrig	Niedrig
			(1,9 %)	(4,3 %)	(8,3 %)	(8,5 %)
3a	> 5–10 cm	≤ 5	Niedrig	Moderat	Hoch	Hoch
			(3,6 %)	(24,0 %)	(34,0 %)	(57,0 %)
3b	> 10 cm	≤ 5	Moderat	Hoch	Hoch	Hoch
			(12,0 %)	(52,0 %)	(34,0 %)	(57,0 %)
4	≤ 2 cm	> 5	ø^a^	Hoch^a^	–	Hoch
				(50,0 %)		(54,0 %)
5	> 2–5 cm	> 5	Moderat	Hoch	Hoch	Hoch
			(16,0 %)	(73,0 %)	(50,0 %)	(52,0 %)
6a	> 5–10 cm	> 5	Hoch	Hoch	Hoch	Hoch
			(55,0 %)	(85,0 %)	(86,0 %)	(71,0 %)
6b	> 10 cm	> 5	Hoch	Hoch	Hoch	Hoch
			(86,0 %)	(90,0 %)	(86,0 %)	(71,0 %)

Basierend auf ca. 2000 Fällen

– Keine Fälle

Ø Kein relevantes Risiko

^a^Sehr geringe Fallzahl

*Die derzeit gängige Auszählung der Mitosen erfolgt in 5 mm^2^

## Pathogenese

Pathogenetisch stellen Mutationen in den **Rezeptortyrosinkinasegenen**

Rezeptortyrosinkinasen

*KIT* oder alternativ *PDGFRA* in 80–85 % aller GIST die größte Subgruppe dar. Bis zu 75 % aller GIST weisen als treibende genomische Alteration eine **aktivierende Mutation**

aktivierende Mutation

im *KIT*-Gen oder in etwa 10–15 % der Fälle im sehr verwandten *PDGFRA*-Gen auf. Die jeweiligen Frequenzen in den verschiedenen Genabschnitten von *KIT *und *PDGFRA* finden sich in Tab. 2 [8].

**Table 2:** 

Genabschnitt	Häufigkeit (%)
*KIT*-Exon 8	0,15
*KIT*-Exon 9	9,33
*KIT*-Exon 11	59,86
*KIT*-Exon 13	1,85
*KIT*-Exon 17	1,70
*PDGFRA-*Exon 12	1,85
*PDGFRA-*Exon 14	0,59
*PDGFRA-*Exon 18	13,99
Non KIT/Non *PDGFRA*	10,58

### Merke

Gastrointestinale Stromatumoren sind am häufigsten im *KIT*-Gen gefolgt vom *PDGFRA*-Gen mutiert.

Dabei handelt sich um sporadische, nur im Tumorgewebe nachweisbare Mutationen, deren genomische Lokalisation und Art vermutlich prognosebestimmend und zudem hoch relevant für ein Therapieansprechen auf die etablierte Erstlinientherapie mit dem Tyrosinkinaseinhibitor **Imatinib**

Imatinib

sind. Die *KIT-* und *PDGFRA-*Mutationen werden schon in sehr kleinen Frühformen (sog. Mikro-GIST, bis 1 cm groß) nachgewiesen, was auf ein frühes pathogenetisches Ereignis hindeutet. Ein Teil dieser kleinen GIST wird insbesondere mit steigendem Alter inzidentell als Zufallsbefund z. B. bei tumorbedingten Magenresektionen oder in Autopsiemägen gefunden. Da die Inzidenz der Mikro-GIST viel höher ist als die der klinisch relevanten, ist anzunehmen, dass weitere onkogenetische Ereignisse für den weiteren Tumorprogress vonnöten sind [9, 10, 11].

Die verbleibenden 10–15 % ohne nachzuweisende Mutation in *KIT *oder *PDGFRA* wurden früher auch als sog. Wildtyp-GIST bezeichnet [12, 13]. Mittlerweile zeigt sich, dass eine weitere große Gruppe der GIST ohne *KIT- *oder *PDGFRA-*Mutation stattdessen einen Defekt im Succinatdehydrogenasekomplex (SDH) aufweist. In einem Teil der Fälle liegen ursächlich **inaktivierende Mutationen**

inaktivierende Mutationen

in der Keimbahn vor, daneben kann der SDH-Komplex auch **epigenetisch inaktiviert** werden. Schließlich sind **Keimbahnmutationen**

Keimbahnmutationen

deutlich seltener auch im *Neurofibromatose-Typ 1*-Gen, in *KIT* und *PDGFRA* beschrieben. Außerdem existieren eine kleine Fallserien mit sporadischen Mutationen im RAS/RAF-Signalweg (*BRAF, KRAS, HRAS, NRAS*) sowie kasuistisch Mitteilungen über Translokationen mit verschiedenen Fusionstranskripten [14].

### Sporadische *KIT*-Mutationen stellen die häufigsten Alterationen in GIST dar

Die* KIT*-Mutationen wurden 1998 erstmalig von Hirota et al. in einer kleinen Fallserie präsentiert [15]. Seither wurden zahlreiche Fallserien publiziert, die auf ein besonders **breites Mutationsspektrum**

breites Mutationsspektrum

in dieser GIST-Gruppe hinweisen. Mittlerweile wurden bei zunehmend eingesetzter Paneldiagnostik folgende **Hotspot-Regionen**

Hotspot-Regionen

für Primärmutationen in *KIT* identifiziert: die Exone 8, 9, 11, 13, 14 und 17. Die Häufigkeitsverteilung in den verschiedenen Regionen ist ganz unterschiedlich, wobei gut zwei Drittel der *KIT*-Mutationen im Exon 11 zu finden sind. Dieser Genabschnitt kodiert die Juxtamembrandomäne, die eine autoregulatorische Region im KIT-Rezeptor darstellt. Das Mutationsspektrum ist breit und wird v. a. von unterschiedlich langen **Deletionen**

Deletionen,

teilweise kombiniert mit Insertionen, und Punktmutationen dominiert. Tumoren mit Deletionen wird ein aggressiveres biologisches Potenzial als solchen mit Punktmutationen und Duplikationen zugeschrieben. Dies gilt insbesondere für Deletionen, die die Codone Trp557 und/oder Lys558 betreffen [16].

Gastrointestinale Stromatumoren mit *KIT-*Exon 9-Mutation sind ganz überwiegend im Dünndarm sowie im Rektum lokalisiert [1], während im Magen nur Einzelfälle beschrieben sind [2, 3, 13]. Zumeist verhalten sich intestinale Tumoren mit dieser Mutation klinisch aggressiv [1, 4], wobei die prognostische Relevanz einer *KIT*-Exon-9-Mutation nach wie vor kontrovers diskutiert wird [17]. Im Rahmen der SSG18-Studie zur Frage der Dauer der adjuvanten Behandlung von GIST konnte gezeigt werden, dass lokalisierte GIST mit einer *KIT*-Exon-9-Mutation mit einem ungünstigen biologischen Verhalten verknüpft sind [18].

**Figure 3:**
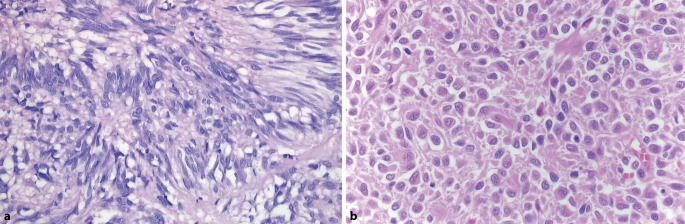

Gastrointestinale Stromatumoren mit einer *KIT*-Mutation besitzen morphologisch mehrheitlich einen **spindelzelligen Phänotyp**

spindelzelligen Phänotyp

(Abb. 3a), zumeist mit sehr monomorphem Aspekt. Eine eher selten zu beobachtende **epitheloide Erscheinungsform**

epitheloide Erscheinungsform

KIT-mutierter GIST geht häufiger mit einer hohen Zellularität und gesteigerter Proliferation einher, zudem kommen solche Phänotypen häufiger in Rezidiven unter TKI-Therapie vor [19].

#### Merke

Das Mutationsspektrum in *KIT* Exon 11 ist breit, der Mutationstyp prognoserelevant.

### *PDGFRA*-mutierte GIST sind die zweitgrößte Gruppe von GIST mit sporadischer Mutation

Seltener als GIST mit *KIT*-Mutationen sind *PDGFRA*-mutierte GIST; sie machen vermutlich 10–15 % aller Fälle aus. Mehrere größere Untersuchungen zeigen übereinstimmend, dass es sich offenbar um eine besondere Subgruppe mit differenter Tumorbiologie handelt. Die *PDGFRA*-mutierten GIST sind fast ausschließlich im Magen zu finden und mehrheitlich gering proliferationsaktiv. Häufiger kommt es bei diesen Tumoren zu einer regressionsbedingten **zystischen Transformation**

zystischen Transformation.

Seltener als bei KIT-mutierten GIST werden bei diesen Fällen ungünstige klinische Verläufe beobachtet.

Ganz gehäuft zeigen die GIST einen epitheloiden (Abb. 3b) oder gemischt **spindelzellig-epitheloiden Phänotyp**

spindelzellig-epitheloiden Phänotyp,

während dieses morphologische Merkmal bei *KIT*-mutierten GIST seltener anzutreffen ist. Es können perinukleäre oder perizelluläre Höfe vorkommen, die **Schrumpfartefakte**

Schrumpfartefakte

darstellen. Die immunhistochemische KIT-Rezeptor-Expression ist häufig nur schwach oder nur fokal nachzuweisen, weshalb ein wesentliches diagnostisches Kriterium von GIST fehlt [20]. Gerade in dieser Gruppe erweist sich der noch sensitivere GIST-Marker DOG1 als hilfreich. Auch der Nachweis von CD34 stützt die GIST-Diagnose. Typisch für *PDGFRA*-mutierte GIST ist außerdem das Auftreten von **mehrkernigen Tumorriesenzellen**

mehrkernigen Tumorriesenzellen,

die teilweise an „flower cells“ erinnern.

### Seltene sporadische Mutationstypen bei GIST

Sehr selten treten alternative Mutationen in *BRAF *[21] oder noch seltener in *KRAS, HRAS* oder *NRAS* auf [22] auf. Diese sind bevorzugt im Dünndarm anzutreffen und weisen einen spindelzelligen Phänotyp auf. Hierzu und zu ebenfalls äußerst seltenen Translokationen gibt es eine umfassende Übersicht von Brcic et al. [14].

### Familiäre und syndromale GIST

Gastrointestinale Stromatumoren mit familiärer Häufung und hereditärer Genese sowie syndromale Formen präsentieren sich zumeist in Kombination mit anderen Tumorentitäten. Zwischen dem Auftreten der verschiedenen Tumorentitäten können große Zeitintervalle liegen, was die Einordnung als syndromale Erkrankung deutlich erschweren kann. Bei Erwachsenen kommen derartige GIST in etwa 10–15 % der Fälle vor, bei Kindern und jungen Heranwachsenden hingegen sind dies bis zu 85 % [23]. Das frühzeitige Erkennen einer derartigen Prädisposition hat erhebliche Konsequenzen für die Therapie aber auch für die Vor- und Nachsorge. Eine hereditäre Genese ist dringend zu vermuten, wenn in der **Familienanamnese** der Patientin/des Patienten mindestens eine weitere erkrankte Person bekannt ist. Erschwert wird die Herstellung eines Zusammenhangs dadurch, dass die diagnostische Einordnung dieser seltenen Tumorerkrankungen medizinisch nicht versierten Familienmitgliedern häufig nicht vorliegt.

### Hereditäre GIST bei *KIT*- oder *PDGFRA*-Keimbahnmutation

Bislang sind über 50 Familien weltweit bekannt, die eine Keimbahnmutation in *KIT *oder in *PDGFRA* aufweisen und bei denen zumindest 2 Familienmitglieder von einem oder mehreren GIST sowie verschiedenen Begleiterkrankungen betroffen sind. Die ganz überwiegende Mehrzahl der Familien weist in der Mutationsanalyse eine heterozygote Keimbahnmutation im *KIT*-Gen auf, während bislang weniger als 10 Familien mit analoger *PDGFRA*-Mutation beschrieben wurden [24]. Bei erstgenannter Konstellation kann auch von einem *KIT*-Mutationssyndrom, bei Letzterer von einem *PDGFRA*-Mutationssyndrom gesprochen werden.

#### *KIT*-Mutationssyndrom

Die Patienten mit einer Keimbahnmutation in *KIT* haben zumeist eine Keimbahnmutation im **Exon 11**

Exon 11

des *KIT*-Gens, also dem Genabschnitt, der auch bei sporadischen GIST am häufigsten betroffen ist (65–70 %; [25]). Vordergründig sind **Punktmutationen**

Punktmutationen

zu finden, nur selten treten stattdessen Deletionen auf (< 10 %), die zumeist nur ein Codon betreffen. Bei sporadischen GIST mit *KIT*-Exon-11-Mutationen kommen hingegen häufig längere Deletionen vor, was zu ihrer zumeist höheren Aggressivität beitragen mag [26]. Als Begleiterkrankungen und mit unterschiedlicher Frequenz je nach Mutationstyp treten in den syndromalen Fällen zusätzlich syn- oder metachron proliferative Prozesse in Zelltypen auf, die den KIT-Rezeptor exprimieren und bei einer Keimbahnmutation in *KIT* ebenfalls aktiviert werden können. Hierzu gehören die **interstitiellen Cajal-Zellen**

interstitiellen Cajal-Zellen

(ICC) in der Muscularis propria des gesamten Gastrointestinaltrakts, die auch als GIST-Vorläuferzellen angesehen werden, sowie Mastzellen und Melanozyten. Entsprechend kommt es bei einem Teil der Patienten zu einer **Dysphagie**

Dysphagie

durch Hyperproliferation der ICC, die sich bandförmig oder mikronodulär zwischen glatter Längs- und Ringmuskulatur des tubulären Gastrointestinaltrakts vermehren. Diese stellen zugleich den Ursprung weiterer GIST bei den betroffenen Patienten dar, die nicht selten als Metastasen gedeutet werden. Die Mastzellvermehrung äußert sich in einer (zumeist systemischen) **Mastozytose**

Mastozytose,

die Aktivierung der Melanozyten durch **Hyperpigmentierungen**

Hyperpigmentierungen,

die auch an den Schleimhäuten auftreten können. Melanome kommen im Rahmen der syndromalen Erkrankung hingegen nicht vor, wobei sporadische *KIT*-Mutationen in Melanomen keine Seltenheit sind. Keimzelltumoren wie etwa Seminome oder Dysgerminome sind im Rahmen des Syndroms nicht beschrieben, obwohl auch in diesen sporadische *KIT*-Mutationen vorkommen. Auch hämatologische Neoplasien kommen nicht gehäuft vor, obwohl in den jeweiligen Vorläuferzellen der KIT-Rezeptor kräftig exprimiert wird und diese ebenfalls sporadische *KIT-*Mutationen aufweisen können.

#### *PDGFRA*-Mutationssyndrom

PDGFRA hat als **Typ-III-Rezeptortyrosinkinase**

Typ-III-Rezeptortyrosinkinase

große Sequenzhomologien zu KIT; beide Gene liegen unmittelbar nebeneinander auf Chromosom 4 und sind vermutlich durch Duplikation entstanden. Gastrointestinale Stromatumoren mit *PDGFRA*-Mutation unterscheiden sich von *KIT*-mutierten GIST morphologisch, prognostisch und therapeutisch. In den wenigen beschriebenen Familien mit *PDGFRA*-Keimbahnmutation liegen entsprechend der differenten Lokalisation und Funktion von PDGFRA andere Begleiterkrankungen vor. Die Betroffenen entwickeln häufig weitere **mesenchymale Tumoren**

mesenchymale Tumoren

im Gastrointestinaltrakt, z. B. inflammatorische fibroide Polypen, fibröse und lipomatöse Polypen sowie Mischtypen. Gehäuft, aber nicht ausschließlich treten diese im Magen auf. In 2 Familien sind als weitere Besonderheit große Hände beschrieben [27, 28].

### Hereditäre GIST bei Neurofibromatose Typ I

Im Gegensatz zu den oben genannten Syndromen treten die **NF1-assoziierten GIST** ganz gehäuft im Dünndarm auf, wobei auch segmentale und rein intestinale Erscheinungsbilder beschrieben sind. Für die letztgenannte Konstellation wird von **genomischen Mosaiken**

genomischen Mosaiken

ausgegangen. Ansonsten sind die für NF1-Patienten typischen **kutanen Manifestationen**

kutanen Manifestationen

(Neurofibrome, MPNST, Café-au-lait-Flecken etc.) zu verzeichnen. Das lebenslange Risiko eines NF1-Patienten, an einem GIST zu erkranken, soll etwa 7–10 % betragen [29, 30].

Erwähnenswert ist, dass in einer jüngeren Arbeit auch in Patienten ohne typische Stigmata einer hereditären Prädisposition bei sog. Wildtyp-GIST gehäuft Keimbahnmutationen sowohl in *NF1* als auch in *KIT, PDGFRA* und *SDHA* gefunden wurden, sodass bei sog. Wildtyp-GIST eine Tiefensequenzierung unter Einschluss aller bekannten GIST-relevanten Gene empfehlenswert ist.

### Sporadische und hereditäre SDH-defiziente GIST

Der SDH-Komplex besteht aus den 4 Isoformen SDHA, SDHB, SDHC und SDHD und befindet sich als Mitglied des **Krebs-Zyklus**

Krebs-Zyklus

in den Mitochondrien sämtlicher Körperzellen. Bei einer inaktivierenden Mutation in einem der 4 Komplexpartner kommt es zum funktionellen Ausfall von SDH und in der Folge zu einer Akkumulation von Succinat in der Zelle. Über die Induktion einer Pseudohypoxie und einer Aktivierung von Hypoxie-induziertem Faktor 1α (HIF-1α) wird eine Signalkaskade ausgelöst, die pathogenetisch über eine **Hypermethylierung**

Hypermethylierung

zahlreicher Gene nicht nur GIST, sondern auch Paragangliome und pulmonale Chondrome verursachen kann. Bei einer Keimbahnmutation in einem der Gene, die für die Komplexpartner SDHA, SDHB, SDHC oder SDHD kodieren, wird die Erkrankung als **Carney-Stratakis-Syndrom**

Carney-Stratakis-Syndrom

bezeichnet. SDH-Mutationen werden selten auch in sporadischen GIST gefunden, bisher beschrieben für *SDHA* [31, 32].

Im Fall einer somatischen methylierungsbedingten Inaktivierung von SDHC treten GIST, Paragangliome und pulmonale Chondrome auf; das Syndrom wird als **Carney-Triade**

Carney-Triade

bezeichnet und kommt nicht familiär gehäuft vor.

Sowohl das Carney-Stratakis-Syndrom als auch die Carney-Triade betreffen in mehr als 80 % der Fälle junge Frauen. Diese **Geschlechtsprädilektion**

Geschlechtsprädilektion

ist bislang unverstanden. Nicht selten sind Kinder oder Jugendliche betroffen. Charakteristisch sind das Auftreten von **multinodulären Tumoren**

multinodulären Tumoren

in der Magenwand sowie der mikroskopisch typische epitheloide Phänotyp. Im Gegensatz zu anderen GIST-Subtypen neigen diese Tumoren zu **regionären Lymphknotenmetastasen**

regionären Lymphknotenmetastasen

und selbst bei bereits manifesten **Lebermetastasen**

Lebermetastasen

zu einem sehr protrahierten Verlauf. Darüber hinaus kommen selten und vornehmlich bei jüngeren Patienten GIST mit SDHB-Ausfall vor, ohne dass eine *SDH*-Keimbahnmutation oder eine Hypermethylierung des *SDHC*-Promotors nachzuweisen ist. Diese Tumoren werden als SDH-defiziente GIST zusammengefasst. Der Anteil von SDH-defizienten GIST an Magen-GIST beträgt nach neuen Untersuchungen etwa 8 %, mit einer hohen Metastasierungsrate, unabhängig von der Risikoklassifikation nach Miettinen und Lasota 2006 [1]. Trotz der Metastasierung zeigen viele (aber nicht alle) Patienten einen bemerkenswert **indolenten Verlauf**

indolenten Verlauf.

Eine Wirksamkeit von Tyrosinkinaseinhibitoren, insbesondere von Imatinib, wird nicht angenommen. Einige Autoren vermuten, dass **Sunitinib**

Sunitinib

oder Sorafenib als Multityrosinkinaseinhibitoren im Fall eines aggressiveren Verhaltens vielleicht wirksamer als Imatinib sein könnten.

## Die Therapie von GIST hängt vom Mutationstyp ab

Als Goldstandard bei der neoadjuvanten, adjuvanten und additiven Therapie von lokalisierten und metastasierten GIST gilt als Erstlinienbehandlung der Tyrosinkinaseinhibitor Imatinib. Das beste Ansprechen zeigen GIST mit einer *KIT*-Exon-11-Mutation. Demgegenüber ist die Responsibilität von Tumoren mit *KIT*-Exon-9-Mutation schlechter als bei *KIT*-Exon 11-mutierten GIST. Die Analyse der europäisch-australischen Studie EORTC62005 sowie des North American Intergroup Phase III Trial (META-GIST) konnte zeigen, dass Patienten mit einem metastasierten *KIT*-Exon 9-mutierten GIST im Hinblick auf das progressionsfreie und Gesamtüberleben von einer Verdopplung der täglichen Imatinibdosis von 400 mg auf 800 mg profitieren [33, 34]. Diese Beobachtung wurde in die europäischen Guidelines zur GIST-Behandlung aufgenommen [35]. Ein größerer Teil der Patienten unter Imatinibbehandlung entwickelt im Laufe der Erkrankungen Sekundärresistenzen, zumeist in Form zusätzlicher sekundärer KIT-Mutationen, die die Bindung von Imatinib an die Tyrosinkinase behindern. Hier stehen als Zweitliniensubstanz Sunitinib, in der dritten Linie **Regorafenib**

Regorafenib

sowie in der vierten Linie Repritinib zur Verfügung.

Die *PDGFRA*-mutierten GIST weisen als häufigsten Mutationstyp die Punktmutation p.Asp842Val auf, die zu einer primären Resistenz gegenüber Imatinib führt. Diese Punktmutation in der Tyrosinkinase 2 und geht mit einer Daueraktivierung dieser Domäne einher. Eine Imatinibbindung ist aber nur an der inaktiven Konformation des Rezeptors möglich. Andere Punktmutationen und Deletionen im Exon 18 sind hingegen imatinibresponsibel [36]. In der überwiegenden Anzahl der Fälle ist die Tumorbiologie von *PDGFRA*-mutierten GIST günstig, sodass eine adjuvante oder additive Behandlung seltener erforderlich ist. Im Fall eines ungünstigen Rückfallrisikos eines *PDGFRA*-mutierten GIST ist die Gabe der kürzlich zugelassenen Substanz **Avapritinib**

Avapritinib

ggf. hilfreich.

## Fazit für die Praxis

- Die Diagnose von gastrointestinalen Stromatumoren (GIST) ist in der ganz überwiegenden Mehrzahl der Fälle problemlos immunhistochemisch möglich.
- Aufgrund der prognostischen und prädiktiven Relevanz ist die molekulare Typisierung von GIST mit relevantem Rückfallrisiko heute fester Bestandteil des diagnostischen Standards.
- Die Risikoklassifikation von GIST ist unter Berücksichtigung der Lokalisation, der Größe, der Mitosezahl/5 mm^2^ und der Frage einer Tumorruptur möglich.
- Bei mehr als einem GIST eines Patienten (syn- oder metachron) oder mindestens einem GIST in der engeren Verwandtschaft sowie bei Begleiterkrankungen (Mastozytose, Pigmentierungen, Dysphagie) sollte eine hereditäre Disposition ausgeschlossen werden.
- Bei jungen, vornehmlich weiblichen Betroffenen kommt ein Succinatdehydrogenase(SDH)-defizienter GIST in Betracht, entweder syndromal oder hereditär.

## CME-Fragebogen

In welchem Abschnitt des Gastrointestinaltrakts kommen gastrointestinale Stromatumoren (GIST) am häufigsten vor?

Ösophagus

Magen

Dünndarm

Kolon

Rektum

Welcher Genabschnitt im *KIT*-Gen ist am häufigsten von somatischen aktivierenden Mutationen betroffen?

*KIT*-Exon 8

*KIT*-Exon 9

*KIT*-Exon 11

*KIT*-Exon 13

*KIT*-Exon 17

Welches ist der sensitivste immunhistochemische Marker für gastrointestinale Stromatumoren (GIST)?

DOG1

KIT (CD117)

CD34

SDHB

Desmin

Welcher Mutationstyp im Exon 11 des *KIT*-Gens geht besonders häufig mit einer aggressiven Biologie einher?

Punktmutation

Deletion

Insertion

Duplikation

Mehrere Punktmutationen

In welchem der unten aufgeführten Gene sind *keine* Keimbahnmutationen, die zu einer gehäuften Inzidenz von gastrointestinalen Stromatumoren (GIST) führen, beschrieben?

*KIT*

*PDGFRB*

*PDGFRA*

*NF1*

*SDH*

Ein mutationsbedingter Defekt der Succinatdehydrogenase ist in einer Subgruppe von gastrointestinalen Stromatumoren (GIST) pathogenetisch entscheidend. Beurteilen Sie die folgenden Aussagen zu diesem Krankheitsbild. Welche Aussage ist richtig?

Succinatdehydrogenase(SDH)-defiziente GIST treten bevorzugt im höheren Lebensalter auf.

Succinatdehydrogenase(SDH)-defiziente GIST sind v. a. im Dünndarm anzutreffen.

Succinatdehydrogenase(SDH)-defiziente GIST können auch auf einer epigenetischen Inaktivierung beruhen.

Succinatdehydrogenase(SDH)-defiziente GIST zeigen einen spindelzelligen Phänotyp.

Succinatdehydrogenase(SDH)-defiziente GIST sind durch eine besonders aggressive Biologie und ungünstige Prognose gekennzeichnet.

Welcher Parameter ist bei gastrointestinalen Stromatumoren (GIST) *nicht* prognoserelevant?

Größe

Lokalisation

Mitosezahl pro 5 mm^2^

Tumorruptur

Nekrosen

Eine 25-jährige Frau stellt sich mit einem zufällig radiologisch entdeckten Rundherd in der Lunge vor. Histologisch erweist sich dieser als pulmonales Chondrom. Welches Syndrom bzw. welche hereditäre Prädisposition sollte in Betracht gezogen werden?

Carney-Stratakis-Syndrom

Carney-Triade

*KIT*-Mutationssyndrom

Neurofibromatose Typ 1

*PDGFRA*-Mutationssyndrom

Welche Therapie stellt beim metastasierten gastrointestinalen Stromatumor (GIST) mit *KIT*-Mutation derzeit den Goldstandard in der Erstlinie dar?

Regorafenib

Avapritinib

Imatinib

Sunitinib

Pazopranib

In der klinisch-pathologischen Konferenz wird der Fall einer 56-jährigen Patientin besprochen. Bei ihr wurde ein gastrointestinaler Stromatumor (GIST) mit Lebermetastasen festgestellt. Die Onkolog*innen schlagen eine Erstlinientherapie in Form einer verdoppelten täglichen Dosis Imatinib vor. Welcher Mutationstyp liegt in dieser Konstellation am ehesten vor?

Punktmutation in *KIT*-Exon 11

Duplikation in *KIT*-Exon 9

Punktmutation in *PDGFRA* (p.Asp842Val)

Deletion im *KIT*-Exon 11

Punktmutation in *BRAF*
